# Clinical Characteristics and Outcomes of Patients With Rome IV Functional Dyspepsia Who Consume Opioids: A Real‐World Study

**DOI:** 10.1111/nmo.15019

**Published:** 2025-02-27

**Authors:** Mohsin F. Butt, Grace Isherwood, Tilly Lewis‐Lawson, Caterina Sbarigia, Christian Lambiase, Razan N. M. Aburumman, Arkadeep Dhali, Debbie Bush, Tim Card, Maura Corsetti

**Affiliations:** ^1^ NIHR Nottingham Biomedical Research Centre Nottingham University Hospitals NHS Trust and the University of Nottingham Nottingham UK; ^2^ Nottingham Digestive Diseases Centre, Translational Medical Sciences, School of Medicine University of Nottingham Nottingham UK; ^3^ Gastrointestinal Unit, Department of Translational Research and New Technologies in Medicine and Surgery University of Pisa Pisa Italy; ^4^ Department of Internal Medicine Henry Ford Hospital Detroit Michigan USA; ^5^ Academic Department of Gastroenterology Sheffield Teaching Hospitals NHS Foundation Trust Sheffield UK

**Keywords:** disorder of gut‐brain interaction, functional dyspepsia, functional gastrointestinal disorder, opioid

## Abstract

**Introduction:**

The prevalence of opioid use and its impact on healthcare outcomes among patients with Rome IV functional dyspepsia (FD) has not been reported in real‐world clinical practice in the United Kingdom (UK). The primary aim of this study was to study the prevalence of opioid intake among outpatients diagnosed with Rome IV FD. Secondary aims were to determine (A) the differences in phenotype and healthcare resource utilization between patients who consumed opioids versus non‐users, and (B) whether a combination of opioid cessation and a neuromodulator prescription could improve gastrointestinal (GI) symptoms.

**Methodology:**

Data were collected from consecutive patients diagnosed with FD according to the Rome IV clinical criteria in a single tertiary care neurogastroenterology outpatient clinic in the UK between January 2016 and December 2021. Patients who consumed opioids were provided with opioid cessation advice and prescribed a neuromodulator (the intervention).

**Results:**

One hundred and fifty‐six patients were diagnosed with FD and 48 (31%) were taking opioids. In a multivariate logistic regression model (OR, [95% CI]), older age (1.03 [1.004–1.059], *p* = 0.03), depression and/or anxiety (4.2 [1.4–12.5], *p* = 0.01), and chronic pain (4.0 [1.8–8.9], *p* < 0.001) were independently associated with opioid consumption at baseline. At least 44% of patients adhered to opioid cessation advice and, among these persons, 29% reported symptom improvement in response to a neuromodulator. The intervention had a number needed to treat of 5.7 to achieve an improvement in clinical symptoms.

**Conclusion:**

Opioid intake in FD is independently associated with older age, depression and/or anxiety, and chronic pain. Encouraging opioid cessation may be an important strategy in the management of FD.

## Graphical abstract

Approximately one in three patients with Rome IV functional dyspepsia (FD) consume opioids and this is associated with older age, depression/anxiety, and chronic pain. Opioid cessation alongside a neuromodulator improved symptoms in 29% of cases, with a number needed to treat of 5.7, suggesting that this treatment strategy may be beneficial in FD management.

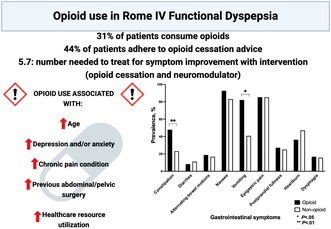

**Summary**

- Approximately one in three outpatients with Rome IV functional dyspepsia consume opioids.
- Opioid use is independently associated with older age, depression and/or anxiety, and a chronic pain condition.
- A combination of opioid cessation and a neuromodulator may have a number needed to treat of 5.7 to achieve an improvement in gastrointestinal symptoms.

## 1 Introduction

The global opioid prescription rate for the management of non‐malignant chronic pain has steadily increased over the past decade [1]. This trend has been associated with increasing opioid‐related morbidity and mortality across North America [2] and Europe [3, 4]. Notably, the gastrointestinal (GI) burden of opioid use appears to be greater in the United Kingdom (UK) than in North America and Europe, as evidenced by findings from the Rome Foundation Epidemiological Survey [5, 6].

Among those with disorders of gut‐brain interaction (DGBI), opioid use is associated with more severe GI symptoms, including more frequent constipation and vomiting, as well as diminished quality of life compared to non‐users [7]. Contrary to best practice recommendations [8], opioids are frequently prescribed to patients with DGBI, even more so than to those with organic GI disease [9].

Rome IV functional dyspepsia (FD) has an estimated population prevalence of 10% in the UK and North America [10]. As with other DGBI, opioids have no therapeutic role in the management of FD [11]. In contrast, there is convincing evidence supporting the use of central neuromodulators, particularly tricyclic antidepressants (TCAs), in the management of DGBI, especially those characterized by abdominal pain [12]. To our knowledge, no UK study has yet addressed the clinical characteristics and healthcare outcomes of patients diagnosed with Rome IV FD who are prescribed opioids, nor the impact of opioid cessation on the treatment response to neuromodulators.

The primary aim of this study was to determine the prevalence of opioid use among outpatients diagnosed with Rome IV FD in a tertiary care neurogastroenterology setting. Secondary aims were to determine: (A) the differences in clinical characteristics and symptomatology between patients consuming opioids versus non‐users; (B) the differences in healthcare utilization (i.e., the number of endoscopic procedures, hospitalizations, and medications) between both groups; and (C) the proportion of patients prescribed neuromodulators who adhered to opioid cessation advice and whether their symptoms improved as a result.

## 2 Methodology

Data were collected from consecutive patients consulted at a tertiary neurogastroenterology outpatient clinic (Queen's Medical Centre, Nottinghamshire, UK) between January 2016 and December 2021.

Patients were newly diagnosed with FD according to the Rome IV clinical criteria, aged ≥ 18 years, and under the care of the senior author (MC). Pregnancy was the only exclusion criterion. A “positive” approach was adopted for diagnosing FD, allowing for a Rome IV FD diagnosis to be made without the requirement to exclude an exhaustive list of similar presentations [13]. Patients were diagnosed with FD without needing to undergo a gastroscopy, provided they tested negative for *H. pylori* and did not present with “red flag” features e.g., anemia, an abdominal mass, or unintentional weight loss. This diagnostic approach aligned with guidance from the British Society of Gastroenterology [8] and is supported by a UK‐based study [14].

Data were collected by three researchers (CS, GI, TLL) from clinical letters authored by MC who used a standardized clinic template to collect information. The standardized clinic template collected data on age, sex, and GI symptoms (constipation, diarrhea, alternating bowel habits, nausea, vomiting, epigastric pain, postprandial fullness, heartburn, and dysphagia), physical and psychological comorbidities, current medications, and previous GI investigations (gastroscopy, sigmoidoscopy, colonoscopy, fecal immunochemical test, abdominal ultrasound, abdominal computed tomography scan, endoscopic ultrasound, abdominal magnetic resonance imaging, GI transit studies, endoscopic retrograde cholangiopancreatography, gastric emptying, anorectal manometry, esophageal manometry, pH impedance, and selenium‐75 homocholic acid taurine testing). This information was collected using binary (yes/no) answers provided by patients. Patient charts were cross‐checked and discrepancies between clinic notes and records were resolved through consensus by MFB and MC.

### 2.1 Opioid Assessment, Down‐Titration, and Treatment with Neuromodulators

Patients were routinely asked whether they consumed opioids. Patients who consumed opioids received verbal and written explanations of the side effects of long‐term narcotic use and the benefits of cessation, along with testimonies from patients who successfully discontinued. Patients were occasionally given the option to be admitted to hospital as an inpatient if they were unable to independently downtitrate their opioid dose in the community.

Typically, the total opioid dose was reduced by 10% per month if patients had been taking opioids for over 1 year [15]. For those who had consumed opioids for a shorter duration of time (i.e., weeks to months), they may have been able to tolerate a faster downtitration in opioid dosage of up to 10% per week. Outpatient consultation letters during follow up were evaluated to determine if patients adhered to opioid cessation advice and, if so, whether they reported any change in their clinical symptoms. Patients who recommenced opioids were included in the group who did not adhere to opioid cessation recommendations.

In line with guidance from the Rome Foundation Working Group on Neuromodulators for DGBI [12], all patients, having already trialed a proton pump inhibitor (PPI) for symptom management, were prescribed a neuromodulator e.g., a TCA or serotonin‐norepinephrine reuptake inhibitor (SNRI). Patients were initiated on 5 mg of either amitriptyline or nortriptyline, tailored to individual circumstances, for a duration of 10–20 days. If tolerated, the dosage was increased to 10 mg, which was reviewed at a 60‐day outpatient consultation. The dosage of TCA was increased incrementally until either clinical response was observed or the maximum dosage (50 mg) was achieved. An augmentation approach, whereby a selective serotonin reuptake inhibitor (SSRI) was added to a TCA, or a TCA was switched to an SNRI, was used in circumstances when patients had no response to single agent therapy.

Clinical response was determined by patients' self‐reported reduction in symptoms associated with FD (i.e., post‐prandial fullness, early satiety and/or epigastric pain).

### 2.2 Statistical Analysis

Continuous and dichotomous variables were expressed as mean (SD) and number (%), respectively. Univariate analyses were performed using the unpaired *t*‐test for continuous variables and the chi‐squared test for categorical variables. Multivariate logistic regression was performed using a forward stepwise approach and co‐variates were determined using domain knowledge and the outcomes of univariate regression models. The following predictor variables were used in the multivariate logistic regression model to identify clinical characteristics associated with opioid use at the initial consultation: (i) age, (ii) depression and/or anxiety, (iii) chronic pain (primary headache disorder, fibromyalgia, lower back pain), and (iv) previous abdominal surgery. For all tests, a two‐sided *p* value < 0.05 was considered significant. Statistical computations were performed using IBM SPSS, version 20 (IBM Corp, Chicago, USA).

This study was approved as a retrospective audit (Nottingham University Hospitals NHS Trust registration number 21‐482C).

## 3 Results

### 3.1 Prevalence of Opioid Use, Differences in Clinical Characteristics, and Healthcare Resource Utilization

One hundred and fifty‐six patients were diagnosed with Rome IV FD, among whom 48 (30.8%) consumed opioids (Table 1).

**TABLE 1:** Differences in demographics, co‐morbidities, and healthcare resource utilization between patients consuming opioids versus non‐users.

Patient characteristic	Opioid users (*n* = 48)	Non‐opioid users (*n* = 108)	*p* ^a^	OR (95% CI) for consuming opioids^b^	*p* ^c^
Demographics					
Sex, female, *n* (%)	39 (81.3%)	88 (81.5%)	> 0.99	1.0 (0.4–2.4)	> 0.99
Age, years, mean (SD)	47.9 (16.1)	40.2 (15.6)	0.005	1.03 (1.01‐1.05)	0.006
FD subtype					
EPS, *n* (%)	29 (60.4%)	63 (58.3%)	0.8	1.1 (0.5–2.2)	0.8
PDS, *n* (%)	2 (4.2%)	8 (7.4%)	0.5	0.5 (0.1–2.7)	0.5
EPS/PDS overlap, *n* (%)	17 (35.4%)	37 (34.3%)	0.9	1.0 (0.5–2.2)	0.9
Co‐morbidities					
Concomitant DGBI, *n* (%)	19 (39.6%)	47 (43.5%)	0.6	0.9 (0.4–1.7)	0.6
Concomitant DGBI, mean (SD)	0.5 (0.6)	0.5 (0.7)	0.6	0.8 (0.5–1.4)	0.6
Depression and/or anxiety, *n* (%)	11 (22.9%)	11 (10.2%)	0.04	2.6 (1.1‐6.6)	0.04
Chronic pain (primary headache disorder, fibromyalgia, lower back pain), *n* (%)	23 (47.9%)	17 (15.7%)	< 0.001	4.9 (2.3–10.6)	< 0.001
Hypermobile Ehlers‐Danlos Syndrome/Hypermobility Spectrum Disorders, *n* (%)	2 (4.2%)	5 (4.6%)	0.9	0.9 (0.2–4.8)	0.9
Atopic condition, *n* (%)	9 (18.8%)	13 (12.0%)	0.3	1.7 (0.7–4.3)	0.3
Neurological disorder, *n* (%)	5 (10.4%)	7 (6.5%)	0.4	1.7 (0.5–5.6)	0.4
Diabetes mellitus, *n* (%)	4 (8.3%)	5 (4.6%)	0.4	1.9 (0.5–7.3)	0.4
Previous abdominal/pelvic surgery, *n* (%)	32 (66.7%)	44 (40.7%)	0.003	2.9 (1.4–6.0)	0.003
Medications and healthcare resource utilization prior to first consultation					
Gastroscopy, *n* (%)	36 (75.0%)	69 (63.9%)	0.3	1.5 (0.7–3.2)	0.3
Colonoscopy, *n* (%)	16 (33.3%)	25 (23.1%)	0.2	1.7 (0.8–3.5)	0.2
GI investigations, mean (SD)	2.4 (1.3)	2.2 (1.4)	0.3	1.1 (0.9–1.5)	0.3
Acid suppression medications (PPI/H2R antagonist), mean (SD)	0.8 (0.7)	0.6 (0.6)	0.03	1.7 (1.04–2.88)	0.04
Anti‐emetics (D2R/5‐HT3R, H1R antagonist), mean (SD)	0.2 (0.4)	0.1 (0.3)	0.01	3.3 (1.3–8.5)	0.02
Laxatives (any), mean (SD)	0.4 (0.8)	0.1 (0.4)	0.004	2.3 (1.2–4.5)	0.01
Hospital admissions (GI‐related), mean (SD)	2.6 (7.0)	1.0 (3.0)	0.06	1.1 (1.0‐1.2)	0.1
Hospital admissions (non‐GI related), mean (SD)	2.7 (4.2)	1.0 (3.2)	0.009	1.2 (1.01–1.31)	0.03

Abbreviations: D2R, dopamine 2 receptor; DGBI, disorder of gut‐brain interaction; EPS, epigastric pain syndrome; FD, functional dyspepsia; GI, gastrointestinal; H1R, histamine receptor‐1; H2R, histamine receptor‐2; PDS, post‐prandial distress syndrome; PPI, proton pump inhibitor; 5‐HT3R, 5‐hydroxytryptamine type 3 receptor.

^a^ *p* value for unpaired *t*‐test (for continuous variables) or chi‐squared test (categorical variables).

^b^ Univariate regression.

^c^ *p* value for univariate regression.

The opioid regimen was available for 30 patients, among whom six (20%) were taking medications pro re nata (PRN), 15 (50%) were taking regular opioids, and nine (30%) were taking both PRN and regular opioids. The mean [SD] dosage of opioid (maximum PRN in 24 h or regular, converted to oral morphine) was 80.2 [68.2] mg. There was no difference in the proportion of females in the opioid versus non‐opioid group (*p* > 0.99), although the mean age was greater among patients who consumed opioids (*p* = 0.005) (Table 1).

Among the 156 patients, 92 (59.0%) were diagnosed with epigastric pain syndrome (EPS), 10 (6.4%) with post‐prandial distress syndrome (PDS), and 54 (34.6%) with EPS/PDS. There was no difference in the prevalence of these three Rome IV subtypes between patients taking opioids versus non‐users (Table 1).

**FIGURE 1:**
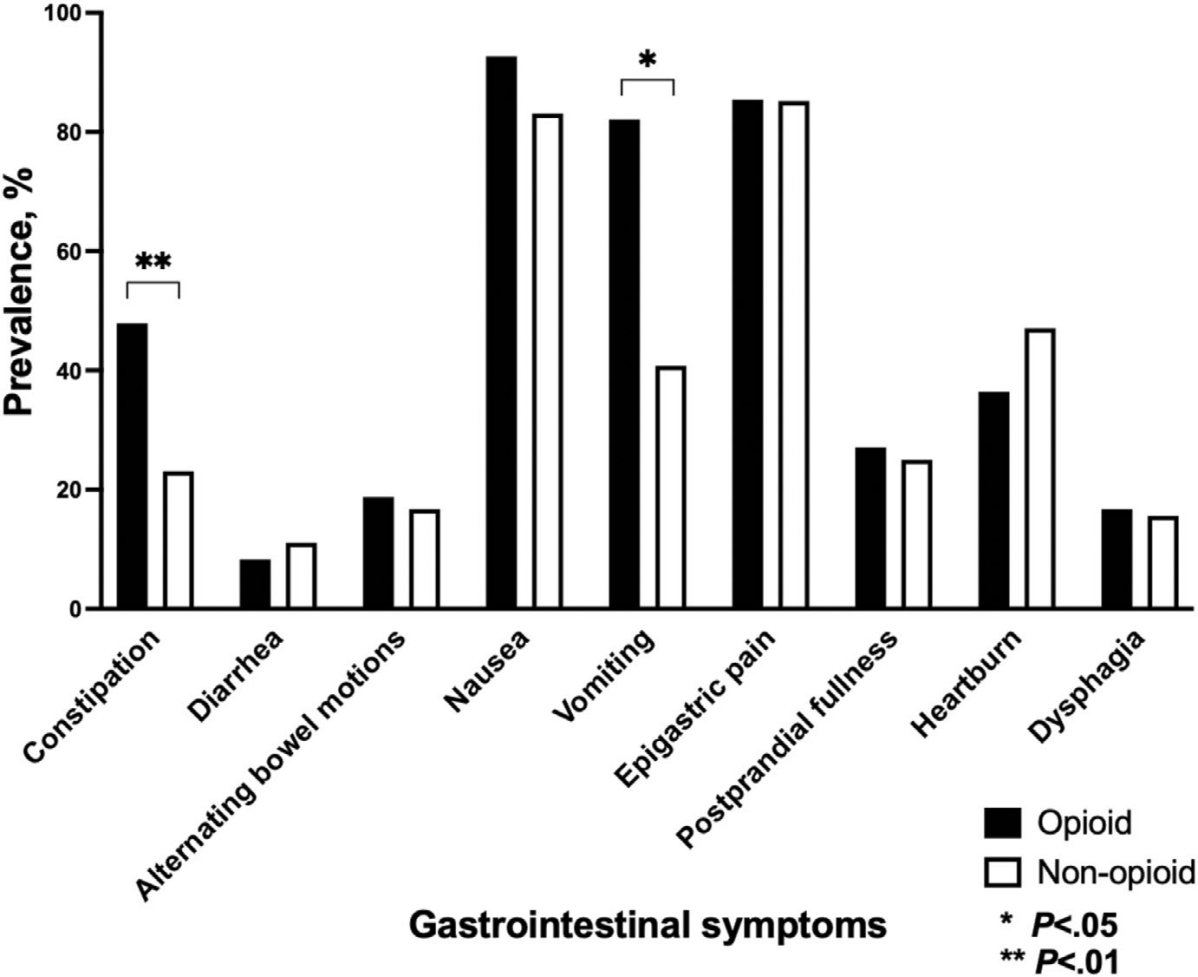
The difference in the proportion of gastrointestinal symptoms reported by patients with functional dyspepsia taking opioids versus non‐users.

A greater proportion of patients in the opioid group reported symptoms of constipation (47.9% vs. 23.1%, *p* = 0.002) and vomiting (82.1% vs. 40.8%, *p* = 0.04). There was no inter‐group difference between the proportion of patients who reported other GI symptoms collected using the standardized proforma (Figure 1). A greater proportion of patients taking opioids were diagnosed with depression and/or anxiety (*p* = 0.04), a chronic pain condition (primary headache disorder, fibromyalgia, lower back pain) (*p* < 0.001), and had undergone previous abdominal/pelvic surgery (*p* = 0.003) (Table 1).

Regarding medications and healthcare resource utilization, the mean number of acid‐suppression medications (*p* = 0.03), anti‐emetics (*p* = 0.01), laxatives (*p* = 0.004), and non‐GI related hospital admissions (*p* = 0.009) were greater among patients taking opioids versus non‐users (Table 1).

In a multivariate logistic regression (OR, [CI]) model, older age (1.03 [1.004–1.059], *p* = 0.03), depression and/or anxiety (4.2 [1.4–12.5], *p* = 0.01), and chronic pain (primary headache disorder, fibromyalgia, lower back pain) (4.0 [1.8–8.9], *p* < 0.001) were independently associated with opioid consumption at baseline (Table 2).

**TABLE 2:** Multivariate logistic regression to identify factors associated with opioid consumption at baseline.

Patient characteristic	Opioids (*n* = 48)	Non‐opioid (*n* = 108)	OR (95% CI) for consuming opioids	*p* value
Age, years, mean (SD)	47.9 (16.1)	40.2 (15.6)	1.03 (1.004–1.059)	0.03
Depression and/or anxiety, *n* (%)	11 (22.9%)	11 (10.2%)	4.2 (1.4–12.5)	0.01
Chronic pain (primary headache disorder, fibromyalgia, lower back pain), *n* (%)	23 (47.9%)	17 (15.7%)	4.0 (1.8–8.9)	< 0.001
Previous abdominal surgery, *n* (%)	32 (66.7%)	44 (40.7%)	2.3 (0.999–5.273)	0.05

### 3.2 Opioid Detoxification and Response to Neuromodulators

**FIGURE 2:**
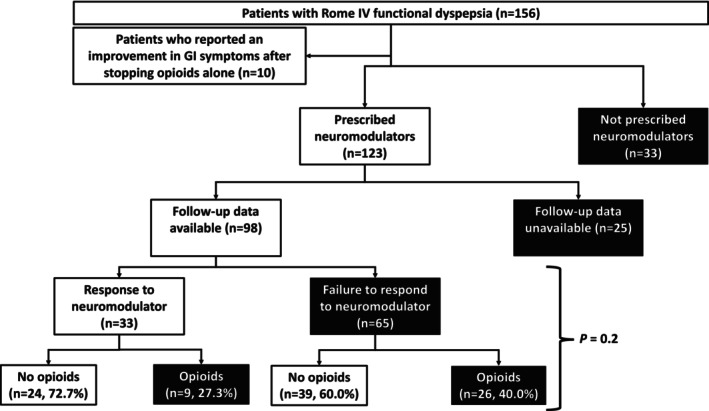
A flowchart detailing the number of patients who responded to neuromodulators, categorized based on opioid and non‐opioid usage. The group not prescribed neuromodulators (n=33) consisted of patients who (A) responded to opioid cessation alone and therefore chose not to receive a neuromodulator (n=5), (B) refused a neuromodulator prescription for unknown reasons, and/or (C) had contraindications to neuromodulator treatment. One hundred twenty‐three patients were prescribed a neuromodulator and follow‐up data were available for 98 of these patients. Among these 98 patients, 33 reported an improvement in FD symptoms after being prescribed a neuromodulator. The improvement in FD symptoms after being prescribed a neuromodulator was not related to opioid intake at baseline (*p* = 0.2). FD, functional dyspepsia; GI, gastrointestinal.

Among the 123 patients who were prescribed neuromodulators, 98 (79.7%) had follow‐up data regarding response to treatment (Figure 2). There was no difference in the mean dosage of amitriptyline or nortriptyline (*p* = 0.8 and *p* = 0.09, respectively) among responders versus non‐responders (Table 3). Similarly, demographics and comorbidity count did not differ between the two groups (Table 3). Opioid use at baseline did not significantly alter the odds of response to neuromodulator therapy, with 27.3% of responders and 40.0% of non‐responders reporting opioid use (*p* = 0.2) (Figure 2). Notably, 10 of the 48 patients (20.8%) who consumed opioids reported an improvement in GI symptoms after discontinuing opioids alone, among whom 5 therefore elected not to take a neuromodulator (Figure 2). Among the 10 patients who reported symptom improvement after discontinuing opioids alone, five patients reported a reduction in abdominal pain (one reported complete resolution of abdominal pain after opioid discontinuation), three patients reported an improvement in constipation, and two patients reported a reduction in nausea and vomiting (one reported complete cessation of vomiting).

**TABLE 3:** Differences in the gastrointestinal (GI) and non‐GI characteristics of patients who responded to neuromodulators versus non‐responders.

Patient characteristic	Responders (*n* = 33)	Non‐responders (*n* = 65)	*p* ^a^	OR (95% CI) for response^b^	*p* ^c^
Demographics					
Sex, female, *n* (%)	24 (72.7%)	55 (84.6%)	0.2	0.5 (0.2–1.3)	0.2
Age, years, mean (SD)	48.3 (17.5)	42.8 (15.9)	0.2	1.0 (1.0‐1.0)	0.1
Medications					
Opioid prescription at baseline, *n* (%)	9 (27.3%)	26 (40.0%)	0.2	0.6 (0.2–1.4)	0.2
TCA prescription in the previous 6 months, *n* (%)	7 (21.2%)	20 (30.8%)	0.3	0.6 (0.2–1.6)	0.3
Final dosage of amitriptyline, mean (SD)	29.4 (13.6)	30.8 (23.4)	0.8	1.0 (1.0‐1.0)	0.8
Final dosage of nortriptyline, mean (SD)	20.8 (6.6)	14.4 (6.2)	0.09	1.2 (1.0‐1.4)	0.1
Acid suppression medications (PPI/H2R antagonist), mean (SD)	0.8 (0.6)	0.7 (0.7)	0.6	1.2 (0.6–2.2)	0.6
Anti‐emetics (D2R / 5‐HT3 receptor, H1R antagonist), mean (SD)	0.1 (0.3)	0.2 (0.4)	0.3	0.6 (0.2–1.8)	0.3
Laxatives (any), mean (SD)	0.3 (0.9)	0.2 (0.6)	0.4	1.3 (0.7–2.3)	0.4
FD subtype					
EPS, *n* (%)	19 (57.6%)	38 (58.5%)	0.9	1.0 (0.4‐2.3)	0.9
PDS, *n* (%)	1 (3.0%)	4 (6.2%)	0.7	0.5 (0.0‐4.4)	0.5
EPS/PDS overlap, *n* (%)	13 (39.4%)	23 (35.4%)	0.7	1.2 (0.5–2.8)	0.7
Co‐morbidities					
Concomitant DGBI, *n* (%)	20 (60.6%)	27 (41.5%)	0.09	2.2 (0.9–5.1)	0.08
Concomitant DGBI, mean (SD)	0.8 (0.7)	0.5 (0.7)	0.1	1.6 (0.9–2.8)	0.1
Depression and/or anxiety, *n* (%)	4 (12.1%)	12 (18.5%)	0.4	0.6 (0.2–2.1)	0.4
Chronic pain (primary headache disorder, fibromyalgia, lower back pain), *n* (%)	11 (33.3%)	19 (29.2%)	0.7	1.2 (0.5–3.0)	0.7
Hypermobile Ehlers‐Danlos Syndrome/Hypermobility Spectrum Disorders, *n* (%)	1 (3.0%)	4 (6.2%)	0.5	0.5 (0.1‐4.4)	0.5
Atopic condition, *n* (%)	4 (12.1%)	6 (9.2%)	0.7	1.4 (0.4–5.2)	0.7
Neurological disorder, *n* (%)	1 (3.0%)	8 (12.3%)	0.1	0.2 (0.0‐1.9)	0.2
Diabetes mellitus, *n* (%)	1 (3.0%)	4 (6.2%)	0.7	0.5 (0.0‐4.4)	0.5
Previous abdominal/pelvic surgery, *n* (%)	17 (51.5%)	35 (53.8%)	0.8	0.9 (0.4–2.1)	0.8

Abbreviations: D2R, dopamine 2 receptor; DGBI, disorder of gut‐brain interaction; EPS, epigastric pain syndrome; H1R, histamine receptor‐1; H2R, histamine receptor‐2; PDS, post‐prandial distress syndrome; PPI, proton pump inhibitor; TCA, tricyclic antidepressant; 5‐HT3, 5‐hydroxytryptamine type 3.

^a^ *p* value for unpaired *t*‐test (for continuous variables) or chi‐squared test (categorical variables).

^b^ Univariate regression.

^c^ *p* value for univariate regression.

**FIGURE 3:**
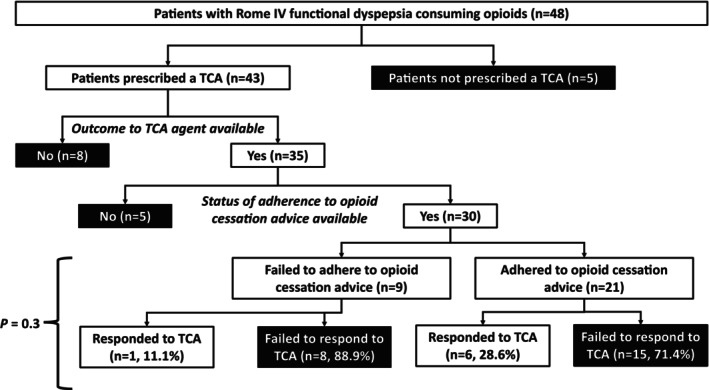
A flowchart detailing the number of patients who responded to TCAs categorized based on adherence to opioid cessation advice. Among 48 patients taking opioids at baseline, 43 were prescribed a neuromodulator. Among these 43 patients, response to neuromodulator treatment and adherence to opioid cessation advice were documented for 30 patients. Among these 30 patients, opioid cessation did not influence response to TCA therapy (*p* = 0.3). TCA, tricyclic antidepressant.

Among the 48 patients who consumed opioids, at least 44% (21/48) adhered to opioid cessation advice (Figure 3). All patients underwent detoxification in the community—one patient was initially considered for inpatient detoxification but ultimately did not undergo this treatment. Among the 30 patients with available data on response to TCA therapy and adherence to opioid cessation advice, the proportion of responders to TCA was not significantly different between those who adhered to opioid cessation advice compared to those who did not (28.6% vs. 11.1%, *p* = 0.3) (Figure 3).

## 4 Discussion

To our knowledge, this is the first study to explore the phenotype and clinical outcomes of outpatients with Rome IV FD diagnosed in routine clinical practice who consume opioids. Approximately 1 in 3 (30.8%) patients who were diagnosed with Rome IV FD in our tertiary care UK neurogastroenterology clinic consumed opioids.

Age was the only demographic variable that differed between persons who consumed opioid versus non‐users, consistent with previous studies demonstrating higher opioid prescription rates with increasing age [16]. A greater proportion of patients in the opioid group were diagnosed with a co‐morbid chronic pain condition i.e., primary headache disorder, fibromyalgia and/or lower back pain. While our study did not evaluate the reasons for opioid use, musculoskeletal, and post‐operative pain are common indications for opioid prescriptions among patients with narcotic bowel syndrome [17]. Our results align with these findings. Opioid intake has consistently been demonstrated to be associated with constipation and vomiting [18], which may partly explain why patients in our study who took opioids consumed a higher number of laxatives, anti‐emetics, and acid‐suppression medications.

There was a signal to suggest that opioid use was associated with increased resource utilization, as evidenced by a higher frequency of hospitalizations. However, the reasons why hospitalizations were more likely to be non‐GI rather than GI related are unclear. Opioids may be inappropriately prescribed to manage chronic pain conditions [19], such as musculoskeletal or neuropathic pain, potentially resulting in complications or exacerbations unrelated to the GI tract that may necessitate hospitalization. Moreover, reverse causality is possible; patients may be discharged from hospital with an opioid prescription, which has been associated with greater post‐discharge healthcare utilization in non‐GI settings [20]. Other measures of resource use (i.e., the number of endoscopic procedures or GI investigations) were not statistically different between both groups.

Only 44% of patients with FD who were prescribed opioids followed opioid cessation advice, which highlights the potential challenge that gastroenterologists face when deprescribing narcotics. We were not able to assess factors associated with drop‐out from the opioid withdrawal process, as likely associations were not collected during routine appointments and hence data were unavailable. Evidence suggests that patients with co‐morbid fibromyalgia and depression have a higher drop‐out rate from the opioid withdrawal process [21] and are more likely to relapse following withdrawal [22]. These are two important factors which should be addressed in future studies assessing opioid downtitration among patients with DGBI.

One major strength of this study is that data were sourced from a single clinic co‐ordinated by one consultant neurogastroenterologist, which guaranteed a standardized technique to assess and diagnose patients with Rome IV FD, including the evaluation of response to neuromodulators. However, we acknowledge that our study has several limitations, especially those that are inherent to any retrospective study conducted in a real‐world setting. Given patients were drawn from one consultant in a single center, the characteristics of our sample may not be entirely representative of the FD population on a national level. Importantly, since data were collected in routine clinical practice, patients did not complete validated GI symptom questionnaires, so the degree of change in the frequency and/or intensity of symptoms with neuromodulator therapy cannot be quantified with absolute precision. To mitigate this, data were collected by multiple investigators who independently reviewed consultation notes and patient records to ensure reliable and consistent data acquisition. Any inconsistencies were resolved through consensus between MFB and MC to enhance reliability. Although we intended to investigate the original indication/s for opioid use, many patients were on long‐term opioid prescriptions and often had difficulty recalling the initial reason for treatment.

An important weakness of our study is its limited power. With a larger, more robust sample, it is possible that the association between baseline opioid consumption and response to neuromodulators, as well as the impact of opioid cessation on treatment response, could reach statistical significance. Were there such a causal relationship, despite poor compliance with opioid cessation, discontinuing narcotics alongside a neuromodulator prescription might have a number needed to treat of 5.7 to achieve an improvement in clinical symptoms. Should the burden of opioids in patients diagnosed with DGBI be as high as our study suggests, then it is possible that an appreciable part of this disease burden may be avoidable via the strict implementation of policies to reduce opioid prescriptions for chronic non‐malignant pain.

In conclusion, 30.8% of patients with Rome IV FD consume opioids, and these individuals tend to be older and have a higher prevalence of chronic pain conditions, depression/anxiety, and a history of abdominal/pelvic surgery compared to non‐users. Healthcare resource utilization, specifically the number of acid suppression medications, anti‐emetics, laxatives, and hospital admissions, is also higher among opioid users. European [23] and North American [24] clinical guidelines currently do not caution against the use of opioids in FD, so our data should prompt a discussion around opioid deprescribing in neurogastroenterology clinics and may help shape future best‐practice recommendations.

## Author Contributions

M.F.B. took the lead in writing the manuscript and performed data analysis. R.N.M.A. and A.D. assisted in data analysis. C.S., G.I., D.B., C.L. and T.L.L. collected data. T.C. and M.C. supervised the project. All authors read and approved the final manuscript.

## Conflicts of Interest

The authors declare no conflicts of interest.

## Data Availability

The data that support the findings of this study are available from the corresponding author upon reasonable request.
